# Analysis of Tweets Containing Information Related to Rheumatological Diseases on Twitter

**DOI:** 10.3390/ijerph18179094

**Published:** 2021-08-28

**Authors:** Adrian Abbasi-Perez, Miguel Angel Alvarez-Mon, Carolina Donat-Vargas, Miguel A. Ortega, Jorge Monserrat, Ana Perez-Gomez, Ignacio Sanz, Melchor Alvarez-Mon

**Affiliations:** 1 Service of Internal Medicine and Rheumatology, Autoimmune Diseases University Hospital “Principe de Asturias”, 28805 Alcala de Henares, Spain; adrian.abbasi@salud.madrid.org (A.A.-P.); aperezalcala@yahoo.es (A.P.-G.); mademons@gmail.com (M.A.-M.); 2 Department of Medicine and Medical Specialities, Faculty of Medicine and Health Sciences, University of Alcala, 28805 Alcala de Henares, Spain; miguel.angel.ortega92@gmail.com (M.A.O.); jorge.monserrat@uah.es (J.M.); 3 Carol Cardiovascular and Nutritional Epidemiology, Institute of Environmental Medicine, Karolinska Institute, 17177 Stockholm, Sweden; carolina.donat.vargas@ki.se; 4 IMDEA-Food Institute, Campus of International Excellence, Universidad Autónoma de Madrid, Consejo Superior de Investigaciones Científicas, 28049 Madrid, Spain; 5 Institute Ramon y Cajal for Health Research (IRYCIS), 28034 Madrid, Spain; 6 Division of Immunology and Rheumatology, Department of Medicine, Emory University, Atlanta, GA 30322, USA; ignacio.sanz@emory.edu

**Keywords:** Twitter, rheumatology, social media, public health, health communication

## Abstract

Background: Tweets often indicate the interests of Twitter users. Data from Twitter could be used to better understand the interest in and perceptions of a variety of diseases and medical conditions, including rheumatological diseases which have increased in prevalence over the past several decades. The aim of this study was to perform a content analysis of tweets referring to rheumatological diseases. Methods: The content of each tweet was rated as medical (including a reference to diagnosis, treatment, or other aspects of the disease) or non-medical (such as requesting help). The type of user and the suitability of the medical content (appropriate content or, on the contrary, fake content if it was medically inappropriate according to the current medical knowledge) were also evaluated. The number of retweets and likes generated were also investigated. Results: We analyzed a total of 1514 tweets: 1093 classified as medical and 421 as non-medical. The diseases with more tweets were the most prevalent. Within the medical tweets, the content of these varied according to the disease (some more focused on diagnosis and others on treatment). The fake content came from unidentified users and mostly referred to the treatment of diseases. Conclusions: According to our results, the analysis of content posted on Twitter in regard to rheumatological diseases may be useful for investigating the public’s prevailing areas of interest, concerns and opinions. Thus, it could facilitate communication between health care professionals and patients, and ultimately improve the doctor–patient relationship. Due to the interest shown in medical issues it seems desirable to have healthcare institutions and healthcare workers involved in Twitter.

## 1. Introduction

Inflammatory and degenerative rheumatic conditions are a large public health problem. More than 21% of US adults were found to have doctor-diagnosed arthritis [1]. Rheumatic diseases may cause discomfort, pain and disability. Among the most common rehabilitation diagnoses (stroke, spinal cord injury, traumatic brain injury, multiple sclerosis, OA, RA, limb loss, and back pain), musculoskeletal conditions, such as back pain and arthritis, have the highest impact on the health care system. This can be explained because of their high prevalence and impact on disability [2]. Osteoarthritis (OA) is the most common rheumatic disease: globally, approximately 300 million people are affected by hip and knee OA, including over 32 million in the United States, which has increased from 21 million in 1990 and 27 million in 2010 [3,4]. The prevalence of OA in different Latin American countries ranges from 2.3% to 20.4% [5]. The global prevalence of hip and knee OA is approaching 5% in adults over 18 years of age. This rate is expected to increase as the population ages and obesity rates increase [6]. The immune mediated inflammatory rheumatic disease prevalence such as rheumatoid arthritis (RA) is estimated to be approximately 800 per 100,000 persons in the United States and European countries [7,8]. The prevalence of systemic lupus erythematosus (SLE) in the United States is 241 cases per 100,000 [9], higher than in the rest of the Americas (Mexico 50, Argentina 58 and Venezuela 78 cases per 100,000) [9] and Spain (210 cases per 100,000) [10]. Furthermore, they can be associated with other pathological conditions such as depression [11]. Recent progress in the understanding of pathophysiology, diagnosis and treatment of rheumatic disease suggests that these diseases might be a topic of interest in current society [12,13,14,15,16].

Social media is used both by general population and the scientific rheumatology community to transmit information of a very diverse nature. This information varies from the expression of their feelings towards the disease or searching for medicines by patients to the diffusion of scientific articles or conferences [17]. The use of social media in rheumatology has increased rapidly in recent years [18]. Social media is used by rheumatology professionals and basic scientists for professional development, networking and education. Due to its ease of access, wide variety of information and convenience (i.e., via mobile applications), social media provides a dynamic medium for medical education, interactive and collaborative learning and networking [19]. Despite the benefits of sharing online health-related material, there are significant concerns regarding the scientific accuracy of available information [20].

Social media brings people to debate and disseminate information, and also influences people’s attitude and health [21]. Twitter is one of the social networking sites more widely used nowadays [22,23]. Via Twitter patients can express themselves and be heard in a more confident, spontaneous and relaxed environment than in their doctor’s office [24]. For this reason, we can find the concerns of patients on Twitter, and get closer to reality in a more exact way than through questionnaires or surveys that are carried out in a medical center [25]. There are papers that already highlight the importance of social networks among patients (patient forums, online blogs, etc.), and this seems to be the way in which the future of rheumatology is progressing [19].

In a recent working meeting, the OMERACT (outcome measures in rheumatology, an independent initiative of international health professionals interested in outcome measure) safety group acknowledged the need to learn more about the safety concerns of treatment in patients with rheumatological diseases, in order to better understand the fears that could lead patients to stop the treatments [26]. In this sense the collaboration of patients is essential, as is the case with all OMERACT initiatives, and it is essential that patients are fully involved in the co-development and co-production of the work. Twitter becomes a useful tool to perceive these perceptions. Although they have been addressed in the context of clinical trials, researching these same fears on Twitter (which are likely to be the same as patients enrolled in a study) can help develop adherence improvement methods.

### Objectives

The aims of this study are the following: (i) Examine the volume and the kind of tweets related to rheumatic diseases. (ii) Describe the users who tweet and their tweets main content. (iii) Investigate which topics generate more engagement on Twitter (measured by the number of retweets and likes generated) as an indicator of user interest in a given topic. (iv) Detect whether there is fake content (posts considered medically inappropriate according to the current medical knowledge). Altogether, it is possible to identify users who tweet about rheumatic diseases and the information they publish and receive. Consequently, it will be possible for health professionals to reach this population by using similar measures already implemented in other areas of medicine [27,28].

## 2. Methods

### 2.1. Study Design and Data Source

In this observational quantitative and qualitative study, we focused on searching for tweets that referred to six rheumatic diseases such as vasculitis, SLE, RA, spondyloarthropathy (SpA), including in turn the term spondyloarthritis, spondylitis, ankylosing spondylitis, psoriatic arthritis (PsA) and reactive arthritis (ReA), OA and Sjögren’s syndrome (SS). Data were obtained in a period of four weeks, spanned from Monday, 25 January to Thursday, 20 February 2020. This time period has at least two months of separation from any major international rheumatology congress. The inclusion criteria for tweets were (1) being public (nonprivate); (2) included in its content any of the diseases mentioned; (3) being posted in English or in Spanish and (4) posted between 25 January and 20 February 2020.

### 2.2. Search Tool and Data Collection

In this study, we used the Twitter Firehose data stream, which is managed by Gnip and allows access to 100% of all public tweets that match a set of “search” criteria (query). In our study, the search criteria were the previously mentioned disease. Tweet Binder, the search engine employed node.js and PHP language that enabled us to analyze tweets in a JavaScript Object Notation (JSON) format, which is used by Gnip.

### 2.3. Content Analysis Process and Creation of the Codebook

A total of 15,250 original tweets were obtained. For the study, a random sample of 10% of the total tweets for each disease was used, with at least 100 tweets (if not, at least 100 tweets were analyzed). This process led to the analysis of 1628 tweets of the six mentioned rheumatic diseases published on Twitter.

**Figure A1:**
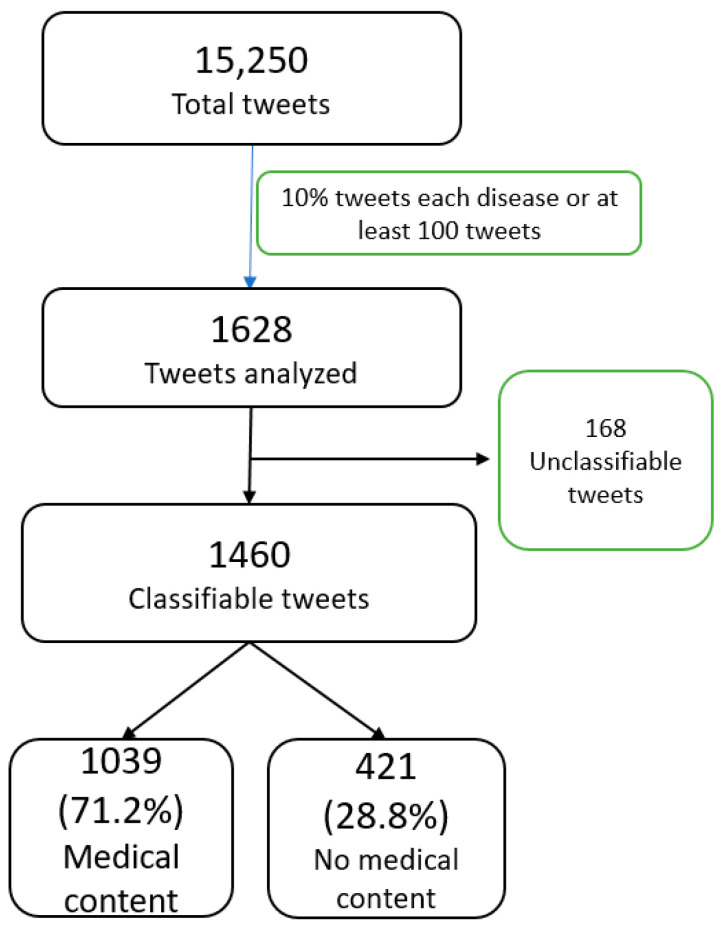
Flowchart.

First, the 1628 selected tweets were scanned by two members of the research team. Next, a codebook was specifically created based on our research questions, our previous experience in analyzing tweets, and the most common tweet topics. Each tweet, depending on the content, was categorized as classifiable or unclassifiable. Differences in categorization and other discrepancies between the raters were discussed with another author until a consensus was reached. Overall, the obtained reliability was higher than 90% for tweet content analysis. We considered a tweet as non-classifiable when its content did not provide enough information (for example, only the nicknames of other users or an external link without further comments appear) or if it was written in a language other than English or Spanish. Note the case of Sjögren’s disease; there were many tweets containing the word “Sjögren” as part of user’s last name and therefore they were non-classifiable. Appendix A (Figure A1) shows a flowchart illustrating the process followed for the analysis of the tweets, along with the number of tweets included and excluded.

In classifiable tweets, the type of user was analyzed first. We distinguish between patient (or family member of a patient or patient association), healthcare personnel (doctors, nurses, physiotherapists, researchers or scientific journals), institutions (official accounts of hospitals, universities, scientific societies or pharmaceutical companies) and others (those that would not be included in the previous mentioned categories such as health blogs, journalists or unidentified users).

Next, it was analyzed whether the tweet is about medical or non-medical content. Among those with medical content, it is analyzed whether the information provided is appropriate or not [29]. When it is not appropriate, we refer to it as fake content, which are those tweets with wrong content or without scientific support, such as the relationship between vaccines and autoimmune diseases or the promotion of therapies not proven to be effective as for example cannabis and gemmotherapy. We also investigate if the intention of the tweet is to broadcast information (disseminate information of scientific interest) and in this case, the type of content (if it talks about the diagnosis or activity disease scales, therapeutics, radiology, pathophysiology, comorbidities, information for patients and others when the topic is different from the previous ones, usually talking about lifestyle such as diet and exercise).

Note two particular cases: the first, the “information for patients” section includes aspects of the disease specifically aimed at patients. It includes written data for patients’ understanding of symptoms, treatment, prognosis, etc. The second, announcements of medical congresses or the offer of research grants or employment opportunities, which have been considered to have medical content but not information dissemination.

When the tweet is in a non-medical content, it is analyzed if the purpose is to seek support (in the form of financial remuneration for a patient, drug collection or even moral support) or if it is different content (a conversation thread, an interview with a patient with a disease, health insurance issue, etc.). The classification criteria we used and examples of tweets according to category are shown in Appendix B (Table A1). Additionally, we analyzed the number of retweets and likes generated by each tweet as an indicator of the user interest in a given topic [30,31].

### 2.4. Ethical Considerations

This study was approved by the Research Ethics Committee of the University of Alcalá and was compliant with the research ethics principles of the Declaration of Helsinki (seventh revision, 2013). This study did not directly involve human subjects and did not include interventions, but instead used publicly available tweets. Nevertheless, we have taken care to not reveal any username and to avoid citing the tweets that could reveal usernames.

### 2.5. Statistical Analysis

Statistics conducted in this work are both descriptive and inferential. All the tweets were statistically analyzed to describe and compare the number of tweets, retweets, and likes depending on the content and other characteristics of the tweet. Retweets and likes are considered as indices for reflecting the interests of the users. We had previously reported the value of retweets in this regard [32,33], so we further calculated the *p* correlations between retweets and likes for all tweets, and by rheumatic diseases, to provide further information.

The statistical difference between the number of tweets generated by different categories was calculated by the Pearson’s Chi-squared test (statistical significance was set at two-sided *p* of <0.05). The statistical significance of the ratios difference between types of rheumatic diseases was obtained by the analysis of variance (ANOVA). We also used simple logistic regressions to calculate the probability of retweeting or liking a tweet of medical content compared to a tweet of non-medical content across the six rheumatic diseases. These results were presented as odds ratio (OR) and their 95% confidence intervals (CI).

These analyses were conducted with the software packages STATA v16 (StataCorp, Madrid, Spain) and SPSS Statistics v23.0.0.0 (IBM Corp, Madrid, Spain).

## 3. Results

### 3.1. Twitter Community Shows a Major Interest in RA and OA Medical Contents

A total of 15,250 original tweets were posted about six rheumatic disorders investigated, vasculitis (*n* = 1180), SLE (*n* = 412), RA (*n* = 4569), SpA (*n* = 962), OA (*n* = 4647), and SS (*n* = 1473), during the period of four weeks, spanned from 25 January to 20 February 2020. For the qualitative analysis of the contents of the rheumatic diseases posted tweets we selected a random sample according to the criteria of including at least a 10% of those of each disease and to have at least a minimum of 100 tweets. We included a total of 1628 tweets. Of these tweets, 168 (10.08%) were considered non classifiable, and the remaining 1460 tweets were included for the qualitative analysis related to the rheumatic disease assessed.

As shown in Table 1, tweets related to RA and OA amounted to 60% approximately (30.3% and 30.6%, respectively) followed by tweets regarding SpA (19.5%). On the other hand, SLE, vasculitis and SS only accounted for 8.2%, 7.5% and 3.9% of the total tweets, respectively. Furthermore, RA and OA tweets accumulated more than the 65% of all retweets and likes generated.

**Figure 1:**
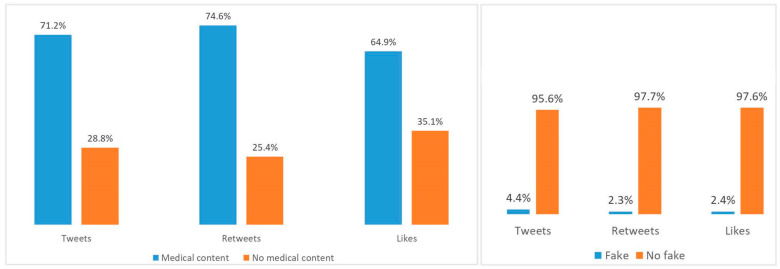
(**Left**): Number of tweets, retweets, and likes generated by medical (blue columns) or non-medical content (orange columns). (**Right**): Number of tweets of medical content, retweets, and likes generated by whether its medical content is fake or not.

Next, we investigated the content of the tweets. First, we categorized the tweets according to their medical and non-medical content according to the stablished criteria. A total of 1039 of the tweets (71.2%) had medical content and 421 non-medical contents (28.8%) (Figure 1). Thus, the medical content was predominant between the tweets related to the rheumatic diseases studied, but with significant differences between them. Medical contents accounted for a 90% of the tweets related to SLE followed by the elevated percentages in those related to OA and RA with the minimum in those posted about SS and SpA (Table 2). Next, we further investigated the specific areas of interest of the tweets with medical content.

Concerning the specific medical contents, it was found that most of the tweets were related to the treatment of the disease (26.5%) followed by information for patients (15.2%). The percentage of tweets with contents referred to the diagnosis (6.8%), comorbidities (6.6%) and pathophysiology of the disease (6.5%) were minor (Table 2).

**Figure 2:**
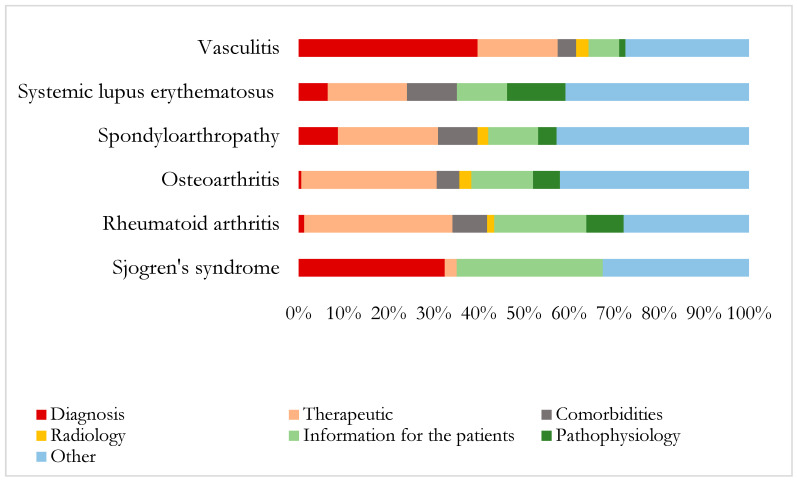
Proportion of the different medical contents among the tweets related to each rheumatic disease. Test χ² *p* < 0.001. Spondyloarthropathy includes: spondyloarthritis, ankylosing spondylitis, psoriatic arthritis, reactive arthritis, and spondylitis. The *p*-value of the Chi-square test for the differences between the percentages of the different medical contents of the tweets related to the rheumatic diseases.

The specific analysis of the type of medical content among the investigated diseases showed a significative heterogeneous distribution (*p* < 0.001) (Figure 2). Contents related to diagnosis were mainly observed in those of vasculitis and SS, and were marginal in those of OA and RA. In contrast, the percentage of treatment related tweets were markedly elevated in those of OA and RA and minimum in those of SS. There were not marked differences in the percentages of tweets related to comorbidities or radiology among the different diseases investigated apart from SS, that lacked contents concerning to comorbidities. The percentage of tweets with pathophysiology contents were clearly higher among those associated with SLE followed by RA and OA and absent in those related to SS. Likewise, information for patients accounted for a relevant percentage of the medical contents regarding to SS and RA, but also was observed in the tweets about the other diseases.

The percentage of tweets with non-medical content was the highest in those related to SpA and lowest in those relating to SLE (Table 3). We found that patient support accounted for a reduced percentage of the tweets with non-medical content that was minimum in those related with OA and maximum in those about SLE.

### 3.2. Types of Users Who Tweet: Different Pattern of Twitter Users Is Found between the Rheumatic Diseases and Type of Contents

The type of users who tweet was investigated. Twitter users were categorized as patients and family members, health professionals, health providers and others that posted the tweets related to the six groups of rheumatic diseases studied (Table 4). Significant differences were found in the percentages of tweets posted by the different users of each group of rheumatic diseases (*p* < 0.001). The number of tweets posted by patients/family members was higher in those related to SS, SpA and vasculitis, and minimum in those regarding SLE. In contrast, the highest number of tweets posted by health professionals were those referred to vasculitis, SLE and OA, and the least, those about SS, SpA and RA (Table 4).

We also investigated the type of tweet content related to rheumatic disease posted by the different categories of Twitter users (Table 5). Patients and family members mostly posted tweets about non-medical contents while health professionals, health institutes as well as unidentified users mainly post tweets with medical contents.

### 3.3. Tweets with Fake Contents Are Mainly Focused on the Treatment of RA, OA and SpA

The scientific appropriateness of the medical contents of the tweets related to the six rheumatic diseases were also analyzed (Table 2). Interestingly, a low percentage of tweets with fake content regarding RA, OA and SpA was observed. Furthermore, it was observed that the content of these fake tweets was mainly focused on the treatment of the diseases. In fact, 5.8% of the tweets related to treatment showed wrong information. There was also a low percentage of tweets with incorrect information about comorbidities and information for the patients about the rheumatic diseases analyzed. The type of user posting fake medical content was also analyzed. It was observed that fake tweets were posted by patients and family members and unidentified users (Table 2). Notably, no fake tweets were encountered among those posted by health professionals or health institutions.

### 3.4. OA and RA Received the Highest Diffusion in Twitter Community and OA Medical Content Generates the Highest Interest

**Figure 3:**
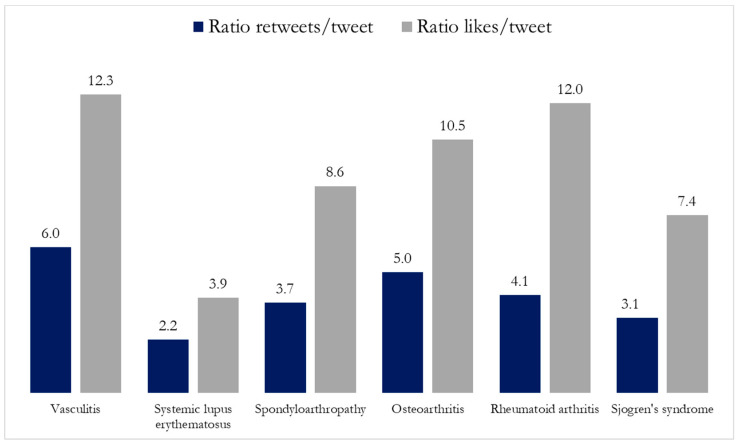
Ratio of retweets and likes per tweet related to rheumatic diseases. Spondyloarthropathy includes: spondyloarthritis. ankylosing spondylitis, psoriatic arthritis, reactive arthritis, and spondylitis. The statistical significance of the ratios difference between types of rheumatic diseases was obtained by the analysis of variance (ANOVA). The *p* value for retweets per tweet is < 0.001 and *p* value for likes per tweet is 0.062.

The interest generated by the tweets about rheumatic disease in the Twitter community was determined as the number of retweets and likes generated [30]. The probability of a post to be retweeted (ratio retweet per tweet) was significantly different between the diseases investigated, being the highest for those of vasculitis and OA and the least for those SLE-related (Figure 3). In regard to the likes, vasculitis and RA related tweets received the highest number of likes per tweet, whereas SLE related tweets had the least number of likes.

Overall, what can be more clearly concluded from Table 6 is that a tweet with medical content is less likely to be liked (OR < 1) than a tweet with non-medical content. Although less consistently, a tweet with medical content seems to be more likely to be retweeted (OR > 1). Whether these observations depend on the type of rheumatic disease to which the tweet refers is difficult to answer with this data because of the lack of statistical power that led to wide confidence intervals. The likelihood of retweeting is significantly higher when the content is medical for OA. In contrast, the probability of liking is lower when the content is non-medical for all diseases except vasculitis and SS.

We also analyzed the response of the Twitter community to medical content fake tweets and we found that they received less retweets and likes (2.3 and 4.9 respectively) than accurate information (4.5 and 9.3 respectively) (Figure 1).

## 4. Discussion

In this study, it is shown that the interest of the Twitter community in rheumatic diseases is centered in OA and RA. The tweets contents are mainly focused on medical contents related to treatments and patient information, but with marked differences between the diseases. Interestingly the accuracy of the medical contents is generally adequate and the probability of being retweeted is higher than those with incorrect information, which are the minor cases.

Currently, the distribution and access to the information in general and to that related to health and disease in particular has been transformed by the increase use of internet and the popularity of social media [34]. Twitter has become a common and effective instrument of health and medical contents diffusion [35,36].

Rheumatic diseases are a wide group of diseases with high prevalence in society that account for a marked morbidity, impairment of quality of life, socioeconomic cost and also mortality [37]. Furthermore, rheumatic diseases are an area of active medical research that have obtained relevant advances in the understanding of the pathogenesis, diagnosis and treatment of the diseases in recent years [12,13,14,15,16]. Furthermore, it has been shown the frequent use of internet by patients with rheumatic diseases [38,39]. Thus, in this study it has been investigated the interest of Twitter users for six relevant diseases with high prevalence such as OA, RA, SpA and SS or with high potential impact on patient survival such as vasculitis and SLE.

The data show that the interest of Twitter community in rheumatic diseases is high according to the number of tweets. This notion is supported by the similarity in the number of tweets referred to the rheumatic diseases and to those previously reported about other prevalent and/or severe diseases [29]. Interestingly, the use of the keywords related to the investigated diseases allowed to classify the tweets, a fact that is indicative of a correct use of the medical term by the Twitter community. This result agrees with a previous study showing the correct use of the word arthritis in this social network [40]. In contrast, an erroneous, random and misuse of terms referred to some medical diseases and conditions has been also previously described [41].

The major number of tweets were about OA and RA. This finding may be explained due to that these diseases are those with a higher prevalence [42,43,44]. In previous studies, it has been also observed the relevance of the prevalence of each disease, as a key factor for representation in the Twitter community [45]. However, other reasons may be also involved in the quantitative relevance of each disease in Twitter such as the clinical and biomedical characteristics of the disease, sociological aspects, and areas of interest of pharmaceutical companies and of medical providers. It has been claimed that the youth of Twitter community may influence the areas of medical interest of the social media users [45].

However, at least in the rheumatic diseases investigated, it does not appear to be a conditioning factor, since OA is a disease with higher prevalence in older population [3,4]. Interestingly, this study is not conditioned by the effects of SARS-CoV-2 pandemic because it was performed during four consecutive weeks between January and February of 2020. The relevance of OA and RA in Twitter community is also supported by the highest reach and impact accounted by both diseases with respect to the others.

Besides the quantitative analysis of the rheumatic diseases in Twitter community, it is highly relevant to identify the areas of interest of the users in these diseases. The results clearly show that Twitter users are mainly interested in the medical aspects of the analyzed rheumatic diseases. The interest in medical contents was variable between the different diseases investigated. It reached the 90% in SLE, and closely followed by those related OA and RA with the minimum percentages in those posted about SS and SpA, but they were always over the 60%. The specific area of the medical contents was mainly focused in therapeutic and information for patients, with a small proportion referring to diagnosis, radiology, comorbidities or pathophysiology. This distribution of medical contents with special interest for therapeutics has been previously identified in non-rheumatic diseases [46,47]. Different non-mutually exclusive reasons may explain this great interest in treatment. First, considering that tweets are limited in space it might be easier to post about treatment related facts rather than explaining the pathophysiology or associated comorbidities. Second, patients may have a higher interest in treatment knowledge than in other aspects of the disease. Third, it may reflect the great interest of business organizations, such as pharmaceutical companies and health providers, to focus on treatment. Fourth, the high number of effective new treatments investigated and introduced in the market for the rheumatic diseases analyzed in the last years. Thus, the multiple therapeutic options available for the patients is an area of interest and is of clinical debate.

Other medical contents received a low percentage of tweets, possibly due to the limited new scientific and medical information generated in these areas. It is interesting to highlight that the diagnosis of the diseases did not receive as many tweets as treatment, despite of the critical importance of diagnostic criteria in Rheumatology [48]. Interestingly, in other diseases in which specific diagnostic tests are critical, the proportion of tweets related to them was reported to be higher [29]. Obtaining the correct information and a proper understanding of the diseases and its treatments by patients appear to be critical in the actual medicine [49,50]. Our findings show a high accuracy in the contents according to the current medical knowledge, as previously observed in other diseases. However, it has been reported a marked percentage of tweets posted by health professional with scientific misleading contents [51]. Interestingly, the precision of the medical contents was total in those posted by health professionals and health institutions, and the fakes were found in those of patients/family members and other sources. Furthermore, we found that the Twitter community used to retweet or like the posts with medical contents, showing a clear ability to select those that are accurate. These results contrast with those reported in urogenital cancer, which shown a higher probability of dissemination of inaccurate or misleading information in social media [52].

These findings encourage health professionals and health institution to participate in providing accurate information in social media. Indeed, it has been already claimed the efficacy of this kind of interventions of health professionals in the prevention and in the treatment of the diseases [53]. In agreement with previous studies in non-rheumatic diseases [45], these results show that non-health professionals (including patients and their families) mainly post tweets with non-medical contents also in the field of rheumatic diseases. This finding supports that Twitter is also used as a friendly place where the community discuss the different matters related to the disease. However, in rheumatic diseases the percentage of tweets showing support was lower than that found in other diseases such as breast cancer, diabetes, depression [54] or HIV [55]. This finding could be explained by the lack of stigma in rheumatic diseases in contrast to the one experienced by patients suffering from other diseases. However, it might be related to under-estimating the severity and life disturbances of these diseases [56,57]. Indeed, patients suffering SpA have expressed frustration on social media in regard to the limited medical consideration of their disease [49].

## 5. Limitations

Although this study improves and expands previous research on the communication of rheumatology in social media, there are still some limitations. First, Twitter may not be reflective of the general population, especially in this field where the patients mean age is higher than that of the most regular Twitter users. Secondly, researchers cannot directly measure clinical outcomes from tweets. Third, the rating process has an inherent degree of subjectivity due to limited information of the context of some tweets, and to the difficulty of accurately verifying the identity of the majority of twitter users. However, these effects were minimized through a robust training before starting the rating process, and by defining the codebook thoroughly. In addition, the analysis was performed by rheumatologists and immunologists.

## 6. Conclusions

Understanding the public view of rheumatic and musculoskeletal diseases is useful to better appraise the perceived demands for clinical care of these patients. It could also help to improve communication between health care professionals and patients and ultimately help to increase the patient’s knowledge of the disease and its management, what could improve the patient’s wellbeing. A trustful doctor–patient relationship constitutes a positive contribution to the therapeutic process. Finally, given the interest raised by medical content posted on Twitter, the involvement of health institutions and health care providers in medical related conversations over social media appears to be desirable as well as the knowledge of reputable online sources that can be recommended to patients.

## Figures and Tables

**Table 1:** Number of tweets, retweets, and likes related to the rheumatic diseases analyzed.

Rheumatic Diseases	Tweets		Retweets		Likes		Correlation Retweets–Likes
	*n*	%	*n*	%	*n*	%	
Vasculitis	109	7.5	656	10.6	1345	9.2	0.70
Systemic lupus erythematosus	120	8.2	264	4.3	472	3.2	0.92
Spondyloarthropathy	285	19.5	1064	17.2	2437	16.6	0.63
Spondyloarthritis	28	1.9	78	1.3	298	2.0	0.29
Ankylosing spondylitis	69	4.7	235	3.8	717	4.9	0.47
Psoriatic arthritis	96	6.6	300	4.9	565	3.9	0.72
Reactive arthritis	8	0.5	1	0.0	1	0.0	−0.14
Spondylitis	84	5.8	450	7.3	856	5.8	0.76
Osteoarthritis	446	30.6	2228	36.0	4669	31.9	0.86
Rheumatoid arthritis	443	30.3	1795	29.0	5307	36.2	0.47
Sjogren’s syndrome	57	3.9	177	2.9	419	2.9	0.98
Total	1460	100.0	6184	100.0	14,649	100.0	0.57

Pearson’s correlation coefficient between retweets and likes, as an index that can be used to measure the degree of relationship. *n*, number.

**Table 2:** Number of tweets with medical contents and of those with fake information related to the rheumatic diseases.

	Total Tweets with Medical Content		Fake Tweets	
	*n*	% of Total Tweets	*n*	% of Total Tweets with Medical Content
Area of Medical Content				
Diagnosis	71	6.8	0	0.0
Therapeutic	275	26.5	16	5.8
Comorbidities	69	6.6	1	1.4
Radiology	20	1.9	0	0.0
Information for patients	158	15.2	2	1.3
Pathophysiology	68	6.5	0	0.0
Other	378	36.4	27	7.1
Total	1039	100.0	46	4.4
Related to Specific Rheumatic Diseases				
Vasculitis	73	67	0	0.0
Systemic lupus erythematosus	108	90	0	0.0
Spondyloarthropathy	171	60	4	2.3
Osteoarthritis	337	75.6	18	5.3
Rheumatoid arthritis	313	70.7	24	7.7
Sjogren’s syndrome	37	64.9	0	0.0
Total	1039	71.2	46	4.4
Type of User that Posted the Tweet				
Patient/family member	32	3.1	3	9.4
Health professional	186	17.9	0	0.0
Health institutes	59	5.7	0	0.0
Other	762	73.3	43	5.6
Total	1039	100	46	4.4

The *p*-value of the Chi square test for the differences between the number of fake tweets by medical content, rheumatic diseases, and type of user were *p* = 0.002, *p* = 0.001 and *p* = 0.001, respectively.

**Table 3:** Number of total tweets with non-medical content, tweets for patient support and tweets about general news by rheumatic diseases.

Rheumatic Diseases	Non-Medical Content					
	Total Tweets		Tweets for Patient Support		General News Tweets	
	*n*	% *	*n*	% ^†^	*n*	% ^†^
Vasculitis	36	33	2	5.6	35	97.2
Systemic lupus erythematosus	12	10	2	16.7	10	83.3
Spondyloarthropathy	114	40	6	5.3	109	95.6
Osteoarthritis	109	24.4	4	3.7	108	99.1
Rheumatoid arthritis	130	29.3	13	10	123	94.6
Sjogren’s syndrome	20	35.1	2	10	18	94.7
Total (*n*)	421	28.8	29	6.9	403	96.0

* Percentage of tweets for the disease over total tweets for that disease (both medical and non-medical). † Percentage of tweets for the disease over tweets with non-medical content for that disease. The *p*-value of the Chi-square test for the differences between the number of tweets for support and the number of tweets about general news by rheumatic disease were 0.27 and 0.13, respectively.

**Table 4:** Rheumatic diseases related tweets classified according to the type of Twitter user.

Rheumatic Diseases	Type of User								TOTAL	
	Patient/Family Member		Health Professional		Health Institutes		Other			
	*n*	%	*n*	%	*n*	%	*n*	%	*n*	%
Vasculitis	27	24.8	30	27.5	4	3.7	48	44	109	100
Systemic lupus erythematosus	5	4.2	24	20.0	6	5	85	70.8	120	100
Spondyloarthropathy	75	26.3	26	9.1	19	6.7	165	57.9	285	100
Osteoarthritis	65	14.6	80	17.9	22	4.9	279	62.6	446	100
Rheumatoid arthritis	77	17.4	45	10.2	20	4.5	301	67.9	443	100
Sjogren’s syndrome	17	29.8	5	8.8	2	3.5	33	57.9	57	100
Total	266	18.2	210	14.4	73	5.0	911	62.4	1460	100

The *p*-value of the Chi-square test for the differences between Twitter users and specific related rheumatic disease is *p* < 0.001.

**Table 5:** Medical vs. non-medical content by type of Twitter user.

Content	Type of User							
	Patient/Family Member		Health Professional		Health Institutes		Other	
	*n*	%	*n*	%	*n*	%	*n*	%
Medical	32	12.0	186	88.6	59	80.8	762	83.6
Non medical	234	88.0	24	11.4	14	19.2	149	16.4

The *p*-value of the Chi-square test for the differences between Twitter users and the content of the posted tweet is *p* < 0.001.

**Table 6:** Probability of retweeting or liking a tweet of medical content vs. non-medical content (reference category) by the related rheumatic disease.

Rheumatic Diseases	Probability of Retweeting			Probability of Liking		
	Num. Tweets with Retweets/with No Retweets		Odds Ratio (95% CI)	Num. Tweets with Likes/with No Likes		Odds Ratio (95% CI)
	Medical Content	Non-Medical Content		Medical Content	Non-Medical Content	
Vasculitis	71/2	36/0	–	66/7	31/5	1.52 (0.45, 5.17)
Systemic lupus erythematosus (SLE)	75/33	11/1	0.21 (0.03, 1.67)	54/54	10/2	0.20 (0.04, 0.96)
Spondyloarthropathy	165/6	112/2	0.49 (0.10, 2.48)	141/30	107/7	0.31 (0.13, 0.73)
Osteoarthritis	315/22	92/17	2.65 (1.35, 5.19)	227/110	90/19	0.43 (0.25, 0.75)
Rheumatoid arthritis	310/3	128/2	1.61 (0.27, 9.78)	224/69	122/8	0.23 (0.11, 0.50)
Sjogren’s syndrome	25/12	12/8	1.39 (0.45, 4.29)	21/16	16/4	0.33 (0.09, 1.17)

Odds ratio and 95% confidence intervals estimated by logistic regression models. NOTE: Those that reach statistical significance are highlighted.

## Data Availability

The data that support the findings of this study are available from the corresponding author upon reasonable request.

## Appendix B

**Table A1:** Examples of tweets by category.

Categories and Definitions	Examples of Classification
**Medical content**	
Appropriate	Chronic Kidney Disease More Common Among Men vs. Women With Ankylosing Spondylitis https://t.co/TxDbFgh7xs#CKD #kidney #kidneydisease (accessed date: 20 February 2021)
Fake tweets	The nutrients in raw cannabis are believed to improve the body’s immune systems functioning and can also be used to treat symptoms of rheumatoid arthritis or lupus while also improving bone metabolism and neural function
**Areas of medical content**	
Diagnosis	Current Issue: Central nervous system vasculitis: advances in diagnosis https://t.co/KydPQw2qbv (accessed date: 20 February 2021)
Therapeutic	International League of Associations for Rheumatology #ILAR recommendations for the management of #PsoriaticArthritis in resource-poor settings. Full text here: https://t.co/pHtlmW7W9O#RheumTwitter https://t.co/7OllpTQydJ(accessed date: 20 February 2021)
Radiology	Prevalence and distribution of MRI abnormalities in the articular cartilage and supporting ligaments in patients with early clinical stage first carpometacarpal joint osteoarthritis.: We used magnetic resonance imaging MRI to evaluate where articular https://t.co/SRI3w4YEsS(accessed date: 20 February 2021)
Pathophysilogy	#mitochondria TL1A/TNFR2-mediated mitochondrial dysfunction of fibroblast-like synoviocytes increases inflammatory response in patients with rheumatoid arthritis via reactive oxygen species generation. https://t.co/tUWP1aa8pB via @xxx(accessed date: 20 February 2021)
Comorbilities	Comorbid chronic diseases common among patients with #osteoarthritis, such as #obesity, #diabetes mellitus and #cardiovasculardisease, have a significant impact on the progression of hip OA, said researchers @xxx(accessed date: 20 February 2021)
Information for patients	“If you are in #pain and you have #psoriasis, #why wouldn’t you go get that checked out by a #rheumatologist? Esp. given everything we know about the #connection between #psoriasis and #psoriatic arthritis.” #WednesdayWisdom from my own #dermatologist https://t.co/By8fjbDmqP @xxx(accessed date: 20 February 2021)
Others	Did you know most fruits and veggies are full of antioxidants, which boost your immune system and may fight inflammation? What to reach for: https://t.co/dEzqS6cI6u(accessed date: 20 February 2021)
**Non-medical content**	
Support for patients	#BlueFamilyPrays Holy Father Please help #Diane09876 who is Battling fibromyalgia & rheumatoid arthritis. She cares for her elderly parents. Her mom has dementia. Cover her with Your Healing Hand & heal her. In Jesus Christ name I pray
News	I’m kind of scared y’all, my doctors think I’m a good candidate for an autoimmune disease called sjogren’s syndrome so they took blood tests today and sent them to the lab, theres no cure and not much treatment either if it comes back positive
